# Risk factors for post-operative intra-abdominal abscess after laparoscopic appendectomy: a case-control study

**DOI:** 10.1186/2197-425X-3-S1-A121

**Published:** 2015-10-01

**Authors:** J Cho, J Lee, K Sung

**Affiliations:** College of Medicine, Department of Surgery, Bucheon St Mary's Hospital, The Catholic University of Korea, Bucheon-si, Republic of Korea

## Introduction

The risk factors for post-operative intra-abdominal abscess (IAA) formation after laparoscopic appendectomy (LA) remain debatable. Some advocate that a perforated appendicitis or Diabetes Mellitus may increase the incidence of post-operative IAA; however, the existing evidence is insufficient.

## Objectives

This study aimed to identify risk factors and non-risk factors for IAA formation in patients receiving LA.

## Methods

From January 2010 to December 2013, all patients who underwent three-port LA and who were histologically diagnosed with appendicitis were included. We classified these patients into two groups according to their development of post-operative IAA and then analyzed the differences between the groups.

## Results

Overall, 1790 patients showed no post-operative complications and were assigned to the non-complication group, whereas 27 patients suffered from IAA postoperatively and were assigned to the IAA group. The incidence of IAA after LA was 1.4% and the only identified risk factor for IAA was dirty fluid collection in the peritoneal cavity observed during the operation. (P < 0.001) On logistic regression analysis of those patients who demonstrated dirty fluid collection, the non-placement of a peritoneal drain had statistical significance for the development of IAA. (P < 0.001)

## Conclusions

In the present study, the presence of dirty fluid collection in the peritoneal cavity rather than the type of appendicitis was demonstrated as a risk factor for the development of post-operative IAA after LA. When such fluid collection is observed, surgeons should consider deploying peritoneal drainage and post-operative antibiotics treatment including anti-anaerobic treatment.
